# Supplementary material for: The YΦ motif defines the structure-activity relationships of human 20S proteasome activators
Source: Nat Commun. 2022 Mar 9;13:1226. doi: 10.1038/s41467-022-28864-x (PMC8907193; doi:10.1038/s41467-022-28864-x)
Supplement: Supplementary file 1 — Supplementary Information [file 41467_2022_28864_MOESM1_ESM.pdf]

Supplementary Information

The YΦ Motif Defines the Structure-Activity Relationships of Human 20S Proteasome Activators

Kwadwo A. Opoku-Nsiah[1,2], Andres H. de la Pena[3], Sarah K. Williams[1,2], Nikita Chopra[1,4,5], Andrej Sali[1,4,5], Gabriel C. Lander[3], Jason E. Gestwicki[1,2*]

Supplementary Fig. 1. a, Pipeline for assay optimization and hit validation of peptide activators of the *h*20S.

Supplementary Fig. 2. Effects of N-terminal modifications on stimulation of *h*20S by peptides.

Supplementary Fig. 3. The structure-activity profile for *h*Rpt5-activated *h*20S differs from the HbYX model.

Supplementary Fig. 4. Dose-dependent stimulation induced by different PAs.

Supplementary Fig. 5. Distinct proteasome substrates report similar SAR for *h*Rpt5-based activators.

Supplementary Fig. 6. Schematic for cryo-EM single-particle data processing.

Supplementary Fig. 7. Cryo-EM metrics for $PA26^{E102A\text{-}Opt5}$-*h*20S complex.

Supplementary Fig. 8. Asymmetric *h*20S open-gate conformation.

Supplementary Fig. 9. Structural evidence implicates termini-mediated gate opening**.**

Supplementary Fig. 10. Binding orientation of reported YΦ motifs align with Opt5.

Supplementary Fig. 11. Biolayer interferometry (BLI) of PA26 activators.

Supplementary Table 1. *h*Rpt5-derived peptides

Supplementary Table 2. Cryo-EM data collection, refinement, and validation statistics

Supplementary Table 3. Measurements associated with open-gate *h*20S cryo-EM structure

Supplementary Table 4. C-terminal sequences of PA200, PAN, Rpt5, Rpt3 and Rpt2 from different organisms identified in UniProtKB.

Supplementary Table 5. List of primers used in this study.

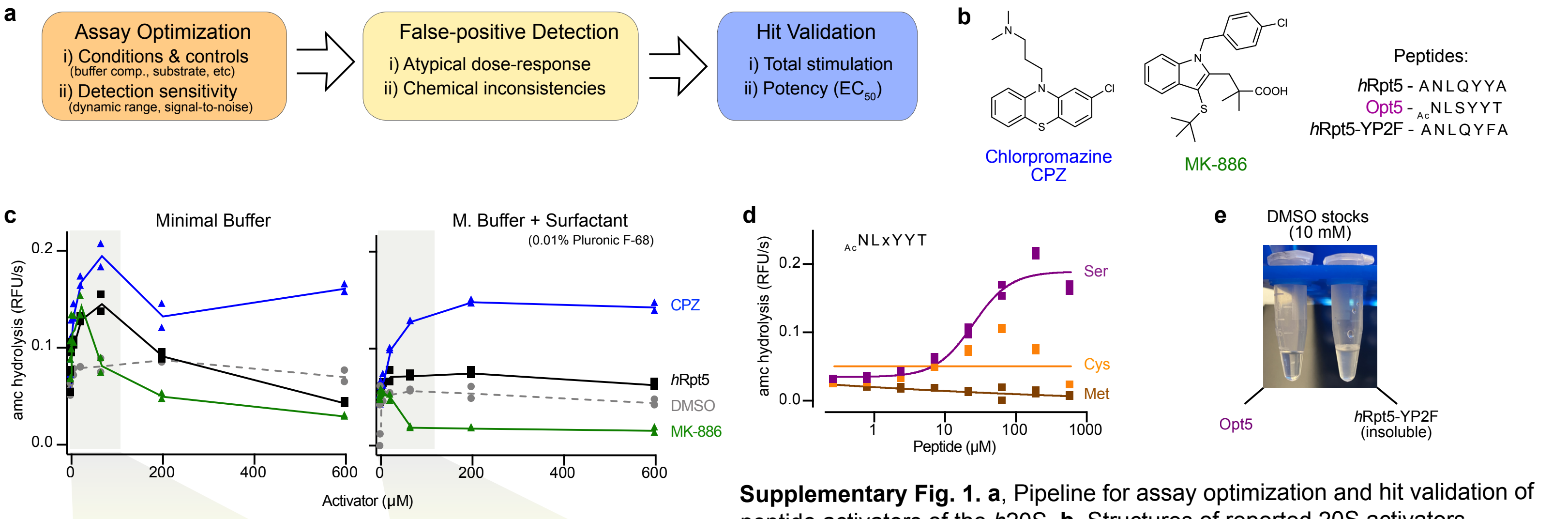

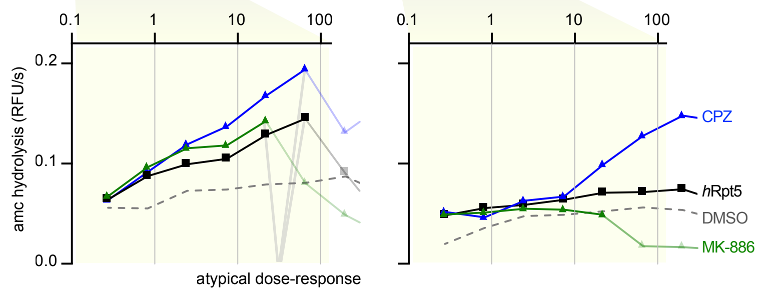

Supplementary Fig. 1. a, Pipeline for assay optimization and hit validation of peptide activators of the *h*20S. **b**, Structures of reported 20S activators and peptide sequences used for assay optimization. **c**, Addition of surfactant (0.01% Pluronic F-68 ®) resolves atypical dose-response curves for *h*20S activators. Representative dose-response curves for small molecule (CPZ, blue; MK-886, green) and peptide (hRpt5, black; Opt5 purple) proteasome activators highlighting inhibition of substrate turnover at high concentrations of activator. **d**, Peptides containing Cys or Met residues exhibit aberrant dose-response curves. Representative dose-response curves for Opt5 with sulfur-containing residues (Cys, orange; Met, brown) at the P4 position. **e**, Noticeably Insoluble peptides in 10 mM DMSO were excluded from this study.

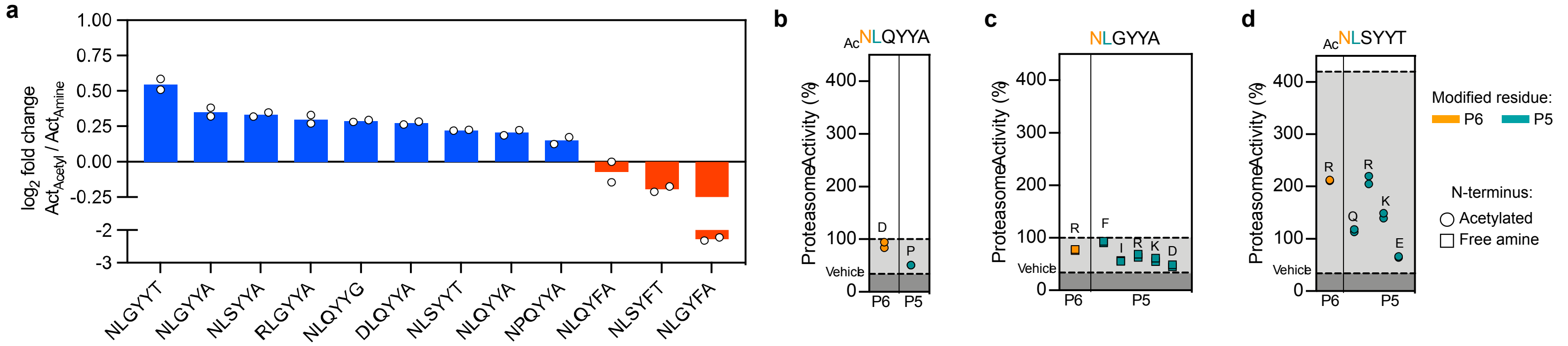

Supplementary Fig. 2. Effects of N-terminal modifications on stimulation. **a**, N-terminal acetylation of *h*Rpt5-derived peptides (250 μM) tends to modestly enhance their stimulatory activity. The effect of N-terminal acetylation is plotted as $\log_2$ fold increase (blue) or decrease (red) in proteasomal stimulation. Reported data is the mean of two independent experiments plotted individually (open circles). **b-d**, Scatterplots of the relative activities of between peptides N-terminally acetylated (circle) or free amine (square) peptides (250 μM) sampling residues at the P6 (orange) and P5 (teal) positions. Activities are normalized to (**b**) $_{Ac}$NLQYYA, (**c**) NLGYYA, or (**d**) $_{Ac}$NLSYYT (dotted lines) and plotted individually (n = 2). Source data are provided as a Source Data file.

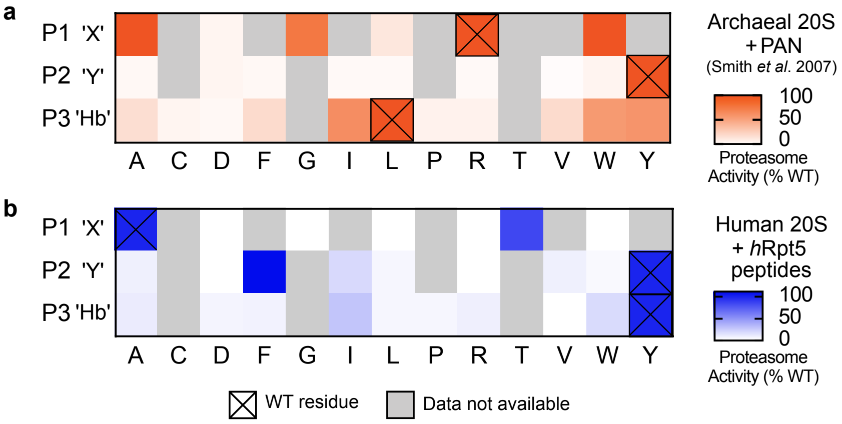

Supplementary Fig. 3. Structure-activity relationships (SAR) for activation of the *h*20S differs from the HbYX model. **a,b**, Comparative heatmaps summarizing SAR of proteasomal stimulation by PAs with different C-terminal sequences for (**a**) archaeal 20S by mutants of the proteasome-activating nucleosidase (PAN) (orange) (Smith *et al*. 2007) and (**b**) human 20S by *h*Rpt5-based peptides in (blue). Residues of WT sequences are denoted with an 'x'. Activity measurements were not determined for residues in gray. **b**, Mutations to PAN from **a** were made to $_{Ac}$NLQYYA. Data are normalized to WT for two independent experiments and plotted as a mean value. Source data are provided as a Source Data file.

a

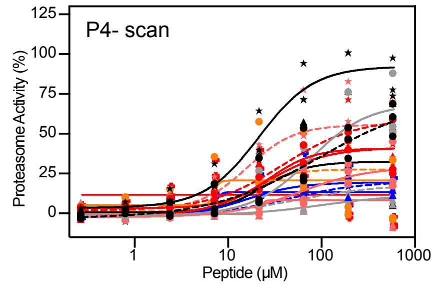

P4 Scan	EC_{50} (µM) Run 1	Run 2	Run 3	Mean (Raw)	Mean (Normalized)
$_{Ac}$NLAYYT	33.2	26.1	43.0	34.1 ± 4.9	27.9 ± 1.3
$_{Ac}$NLDYYT	77.1	76.3	76.9	76.8 ± 0.2	74.9 ± 1.4
$_{Ac}$NLEYYT	N.D.	53.5	83.6	68.5	53.1 ± 3.2
$_{Ac}$NLFYYT	> 600	> 600	> 600	N.D.	> 600
$_{Ac}$NLGYYT	68.7	> 600	20.5	44.6	44.6 ± 2.2
$_{Ac}$NLHYYT	33.9	48.1	15.9	32.6 ± 9.3	25.8 ± 1.5
$_{Ac}$NLIYYT	> 600	> 600	> 600	N.D.	> 600
$_{Ac}$NLKYYT	> 600	> 600	> 600	N.D.	> 600
$_{Ac}$NLLYYT	> 600	> 600	> 600	N.D.	> 600
$_{Ac}$NLNYYT	17.1	21.7	27.9	22.2 ± 3.1	23.8 ± 2.2
$_{Ac}$NLPYYT	62.3	39.4	29.8	43.8 ± 9.6	39.2 ± 1.5
$_{Ac}$NLQYYT	53.6	63.9	32.9	50.1 ± 9.1	46.9 ± 1.5
$_{Ac}$NLRYYT	> 600	> 600	> 600	N.D.	> 600
$_{Ac}$NLSYYT	27.5	25.0	15.0	22.5 ± 3.8	21.4 ± 1.2
$_{Ac}$NLTYYT	17.0	13.1	18.0	16.0 ± 1.5	16.2 ± 1.4
$_{Ac}$NLVYYT	N.D.	14.7	18.0	16.3	16.7 ± 1.6
$_{Ac}$NLWYYT	> 600	> 600	> 600	N.D.	> 600
$_{Ac}$NLYYYT	> 600	> 600	> 600	N.D.	> 600

b

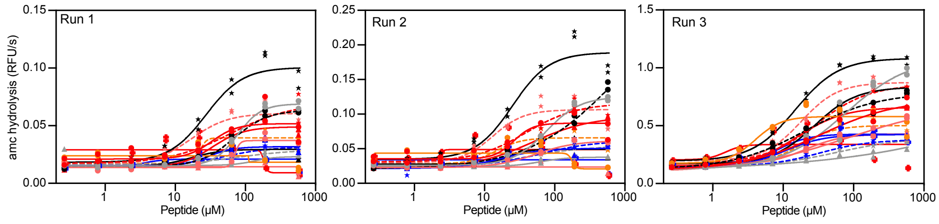

c

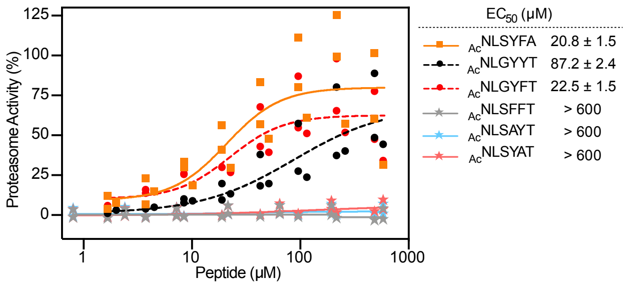

d

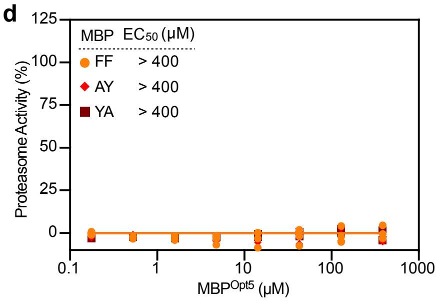

e

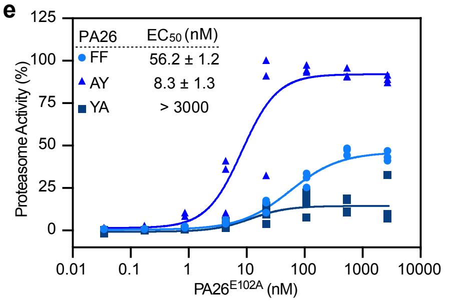

Supplementary Fig. 4. Stimulation of *h*20S by different PAs. **a-c**, Dose-response curves for P4-substituted (**a**,**b**) and other *h*Rpt5-derived (**c**) hexapeptides. **a,c**, Data are normalized to Opt5 ($_{Ac}$NLSYYT) with the mean value plotted for three independent experiments (n = 3). EC_{50} values are reported as means with error reported as s.e.m. **b,** Unnormalized experimental replicates are plotted as means for technical duplicates (n = 2). Data normalization had no significant impact on calculated potencies. **d-e,** Dose-response curves for Opt5-based sequences in MBP (**d**), and PA26 (**e**) constructs. Reported data is normalized to NLSYYT and the mean is plotted for independent experiments (n = 3). EC_{50} values are reported as mean +/- s.e.m. Source Data are provided as a Source Data file.

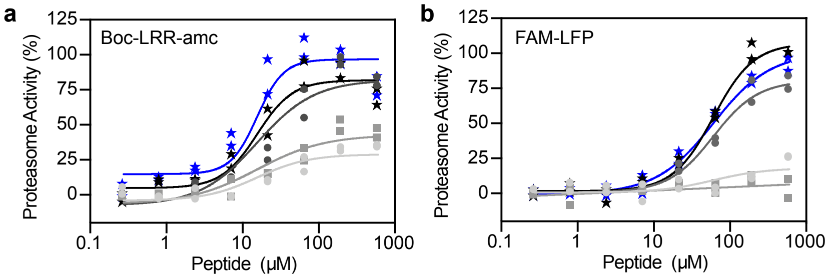

EC$_{50}$ (μM)	LLVY	LRR	LFP
$_{Ac}$NLSYFT	17.3 ± 1.1	9.3	48.5
$_{Ac}$NLSYYT	35.5 ± 1.1	15.3	58.5
$_{Ac}$NLSYYA	65.0 ± 1.1	35.1	72.2
$_{Ac}$NLQYYT	66.0 ± 1.4	42.2	> 600
$_{Ac}$NLQYYA	> 600	47.3	> 600

Supplementary Fig. 5. hRpt5-based activators stimulate the *h*20S similarly using different proteasome substrates. **a,b**, Stimulation of the *h*20S (4 nM) by *h*Rpt5 peptides was assessed with trypsin-targeted boc-LRR-amc (LRR; 20 μM) (**a**) and gate-sensitive nonapeptide FAM-LFP (LFP; 100 nM) (**b**). Data are normalized to $_{Ac}$NLSYFT (**a**) or $_{Ac}$NLSYYT (**b**) and plotted individually (n = 2). Calculated EC$_{50}$ values are reported for LRR and LFP substrates along with LLVY (**Fig. 1f**). Source data are provided as a Source Data file.

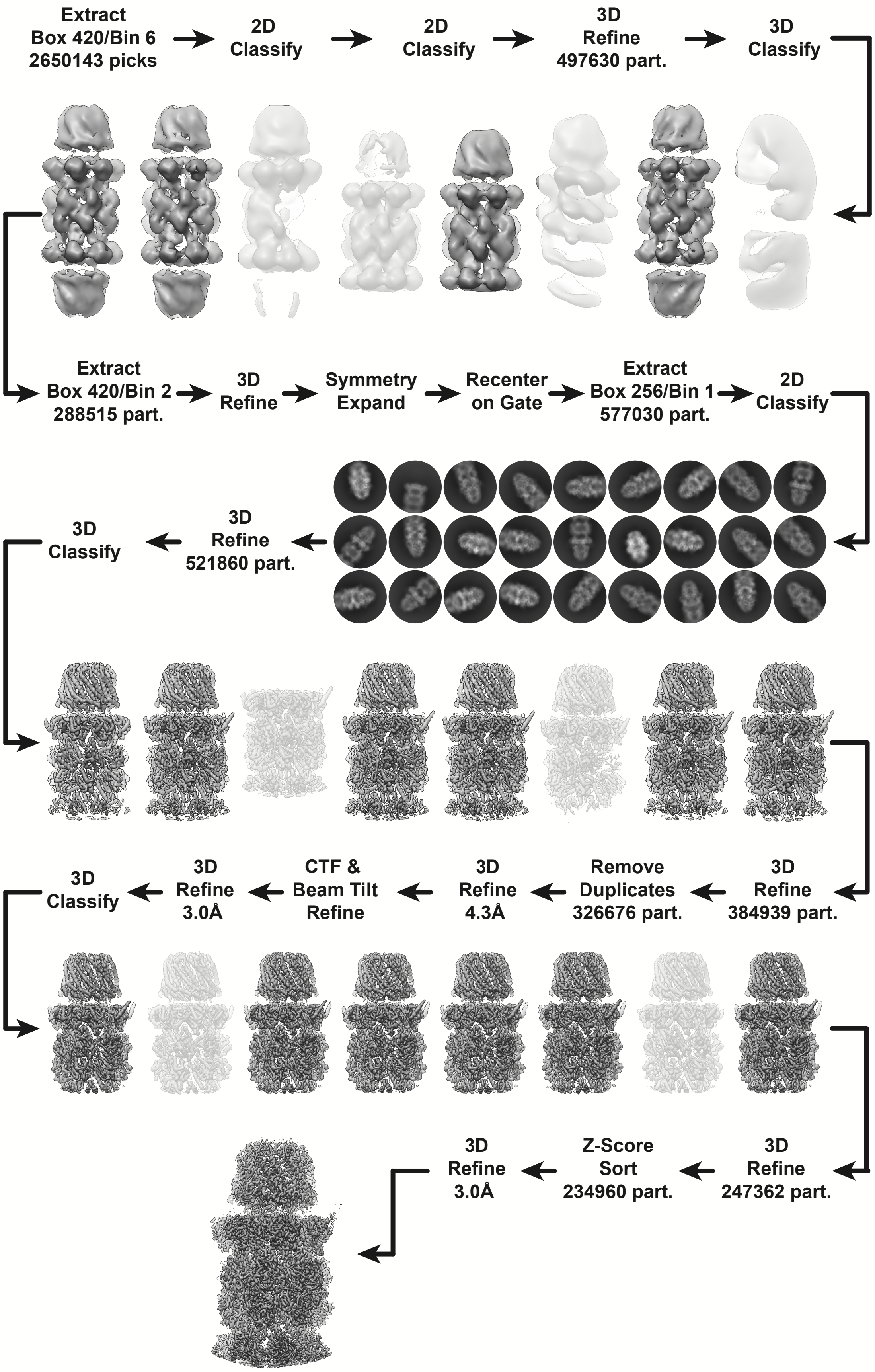

Supplementary Fig. 6. Schematic for cryo-EM single-particle data processing. Low resolution or artefactual reconstructions (transparent) and corresponding particles were excluded from subsequent processing steps, whereas remaining reconstructions (gray) and corresponding particles were utilized. Representative 2D classes are shown in black circles.

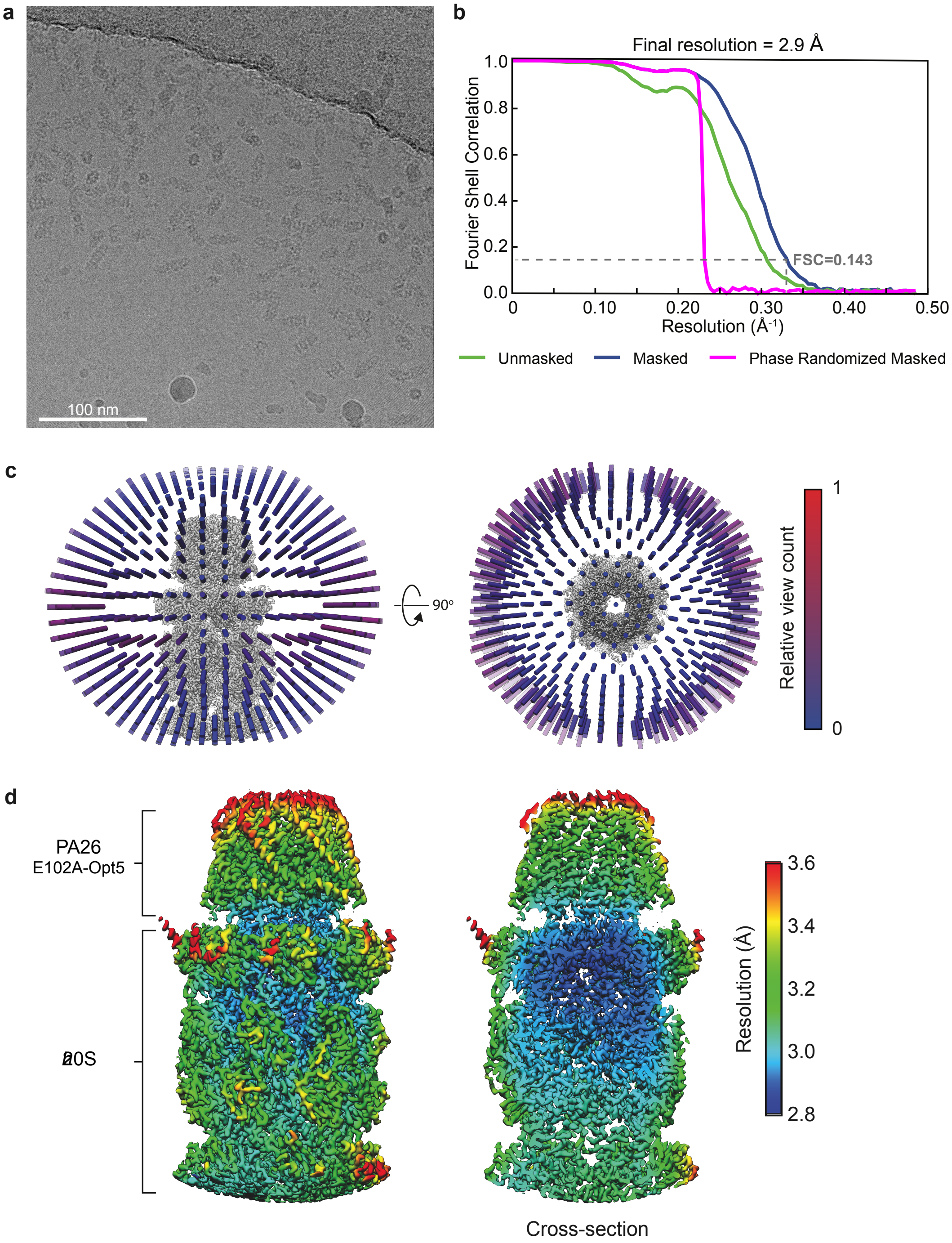

Supplementary Fig. 7. Cryo-EM metrics for PA26$^{E102A\text{-}Opt5}$-*h*20S complex. **a**, Representative micrograph (234,960 particles). **b**, The Fourier Shell Correlation (FSC) curves for the unmasked (green), masked (blue) and phase randomized (magenta) reconstructions. The resolution at 0.143 is indicated by a dashed line. **c**, Distribution of Euler angles displaying the orthogonal view of the reconstruction. The angular distribution is shown as a column whose longitudinal axis aligns with the normal of the corresponding back-projection **d**, Local resolution estimates of the global reconstruction for the complete volume (left) and a coronal cross-section (right). **c,d** Generated and calculated with RELION 3.0.

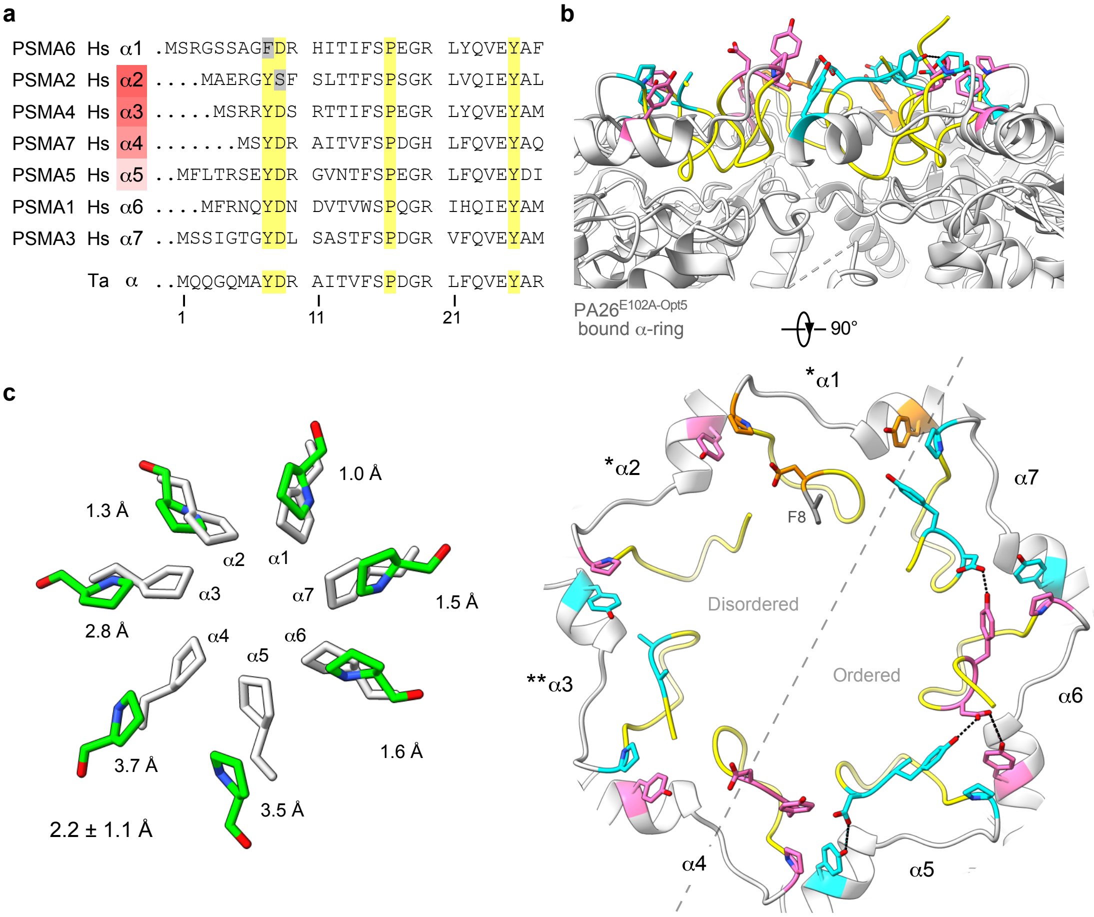

Supplementary Fig. 8. Asymmetric contributions of the *h*20S N-termini in gating. **a**, N-terminal sequence alignment of *T. acidophilum* (Ta) and *H. sapien* (Hs) α-subunits depicting the conserved (yellow) and non-canonical (gray) residues that regulate gating. The α-subunits that occlude the α-annulus are highlighted in shades of red where the intensity corresponds to the relative contributions in gating. The human gene name is accompanied with the systematic nomenclature of the α-subunits. **b**, Cartoon representation of the α-ring depicts the extreme N-termini positioned away from the α-annulus and interactions (black dotted lines) between gating residues (α1, orange; α2, α4, α6, pink; and α3, α5, α7, cyan) of the *h*20S. Single and double asterisks denote α-subunits with unresolved N-termini that either featured non-canonical (*) or canonical gating residues (**), respectively. The overlap between the ordered N-termini and the interacting clusters in a given hemisphere suggest that the non-canonical residues of the human 20S may impedes gate opening. **c**, Radial displacement of αPro17. Individual measurements are reported along with mean ± s.d.

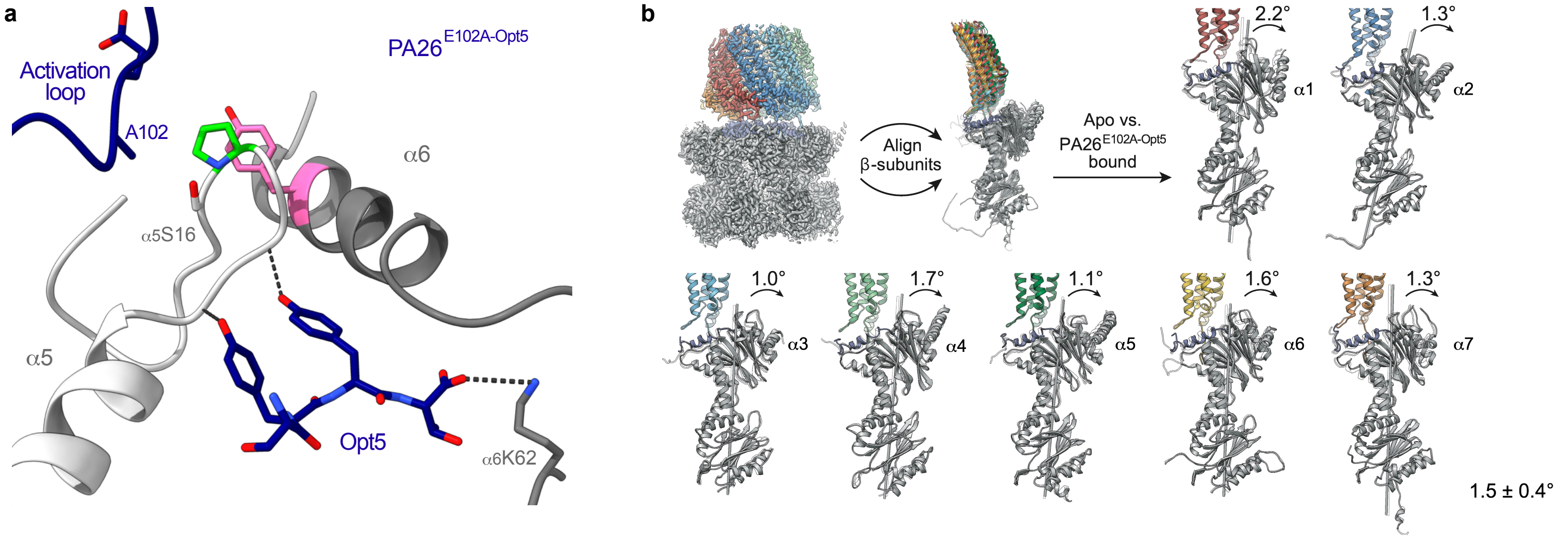

Supplementary Fig. 9. Structural evidence implicates termini-induced gate opening. **a**, Disabled activation loop of PA26$^{E102A-Opt5}$ no longer interacts with αPro17 (green) reverse turn in representative α-pocket (α5/α6-pocket) yet however, C-terminal Opt5 engages residues within reverse-turn loop from the α-pocket. **b**, PA26$^{E102A-Opt5}$ induces rotation of the α-subunits about the axial channel. Individual measurements are reported along with mean ± s.d.

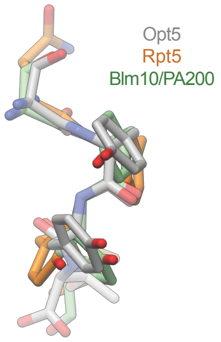

Supplementary Fig. 10. Overlay of bound Opt5 (SYYT, gray) with previously reported structures of docked human Rpt5 (QYYA, orange) (PDB ID: 5GJR [http://doi.org/10.2210/pdb5gjr/pdb]) and yeast Blm10 (SYYA, green) (PDB ID: 4V70 [http://-doi.org/10.2210/pdb4v70/pdb]) depicts a shared docking pose that potentially promotes intramolecular contacts between P2 and P3 side chains.

a

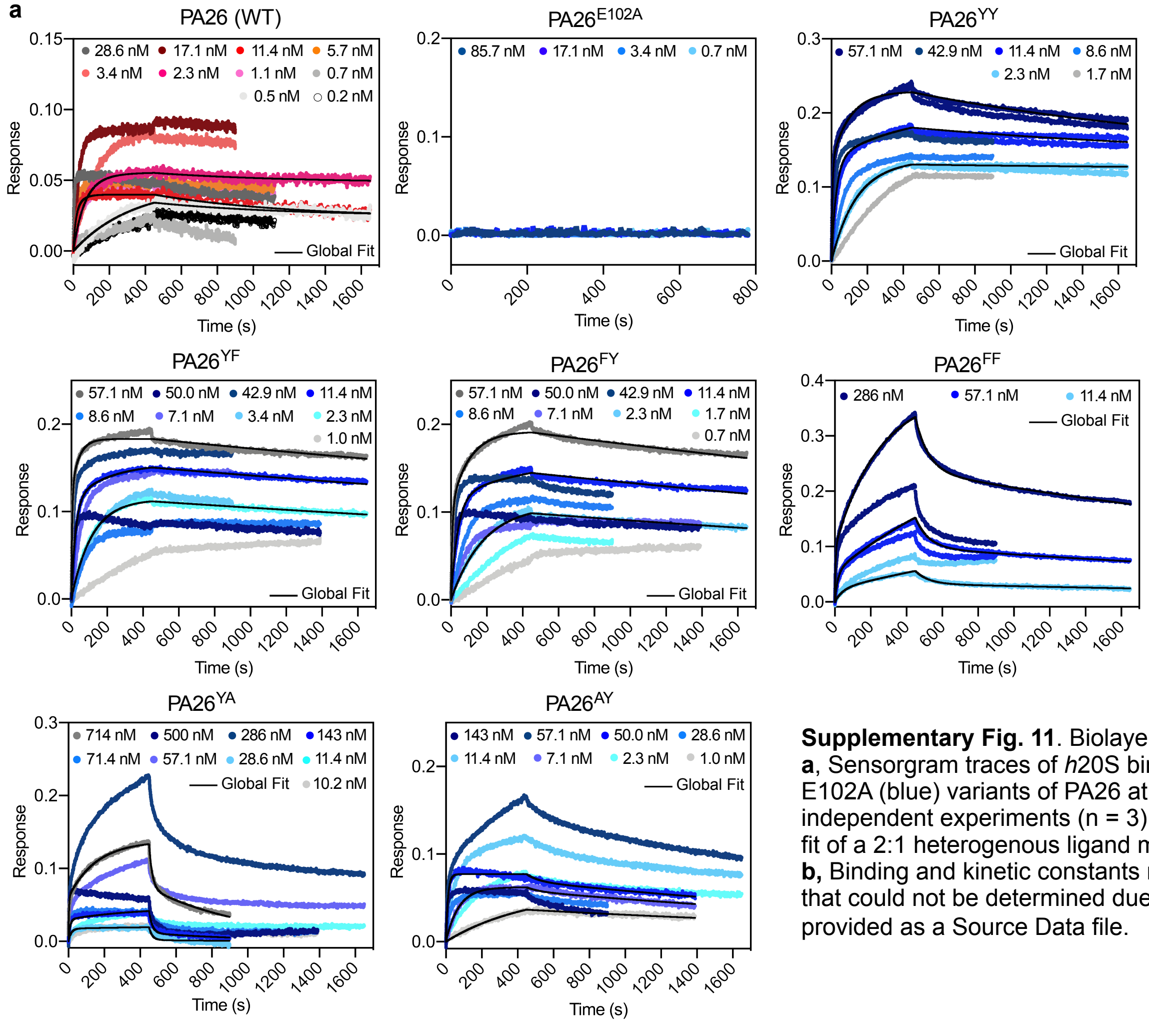

b

PA26	K_d ± s.e.m. (10^{-11} M)	k_{on} ± s.e.m. (10^4 $M^{-1}s^{-1}$)	k_{off} ± s.e.m. (10^{-4} s^{-1})
WT	10 ± 5	710±310	2.9 ±0.8
E102A	> 9000	N.D.	N.D.
$PA26^{YY}$	36 ± 16	49±4	1.6±0.7
$PA26^{YF}$	22 ±10	150±30	2.6±0.5
$PA26^{FY}$	38 ±16	120±60	3.5±1.6
$PA26^{FF}$	3500±1800	1.2±0.3	79±75
$PA26^{YA}$	3600±900	27±26	110±110
$PA26^{AY}$	28±13	150±20	4.0±1.6

Supplementary Fig. 11. Biolayer interferometry (BLI) of PA26 activators. **a**, Sensorgram traces of *h*20S binding to either wildtype (WT, red) or E102A (blue) variants of PA26 at different concentrations for three independent experiments (n = 3). Solid black lines correspond to global-fit of a 2:1 heterogenous ligand model for one representative experiment. **b,** Binding and kinetic constants measured for **a**. N.D. denotes values that could not be determined due to undetectable binding. Source data are provided as a Source Data file.

Supplementary Table 1. List of *h*Rpt5-derived peptides.

ID	Peptide length	N-term Modification	Sequence	Modification
1	7	-	ANLQYYA	-
2	7	-	AALQYYA	NP6A
3	7	-	ANAQYYA	LP5A
4	7	-	ANLAYYA	QP4A
5	7	-	ANLQAYA	YP3A
6	7	-	ANLQYAA	YP2A
7	6	-	NLQYYA	-
8	5	-	LQYYA	-
9	4	-	QYYA	-
10	6	Ac	NLQYYA	-
11	6	Ac	NLQYYA-$CONH_2$	C-term amide
12	6	-	NLQYYD	AP1D
13	6	Ac	NLQYYD	AP1D
14	6	-	NLQYYG	AP1G
15	6	Ac	NLQYYG	AP1G
16	6	Ac	NLQYYL	AP1L
17	6	Ac	NLQYYR	AP1R
18	6	Ac	NLQYYT	AP1T
19	6	Ac	NLQYYW	AP1W
20	6	Ac	NLQYAA	YP2A
21	6	Ac	NLQYDA	YP2D
22	6	-	NLQYFA	YP2F
23	6	Ac	NLQYFA	YP2F
24	6	Ac	NLQYIA	YP2I
25	6	Ac	NLQYLA	YP2L
26	6	Ac	NLQYRA	YP2R
27	6	Ac	NLQYVA	YP2V
28	6	Ac	NLQYWA	YP2W
29	6	Ac	NLQAYA	YP3A
30	6	Ac	NLQCYA	YP3C
31	6	Ac	NLQDYA	YP3D
32	6	-	NLQFYA	YP3F
33	6	Ac	NLQFYA	YP3F
34	6	Ac	NLQIYA	YP3I
35	6	Ac	NLQLYA	YP3L
36	6	Ac	NLQPYA	YP3P
37	6	Ac	NLQRYA	YP3R
38	6	Ac	NLQSYA	YP3S
39	6	Ac	NLQVYA	YP3V
40	6	-	NLQWYA	YP3W
41	6	Ac	NLQWYA	YP3W
42	6	-	NLGYYA	QP4G
43	6	Ac	NLGYYA	QP4G
44	6	-	NLSYYA	QP4S
45	6	Ac	NLSYYA	QP4S
46	6	Ac	NLAYYT	AP1T, QP4A
47	6	Ac	NLCYYT	AP1T, QP4C
48	6	Ac	NLDYYT	AP1T, QP4D
49	6	Ac	NLEYYT	AP1T, QP4E
50	6	Ac	NLFYYT	AP1T, QP4F
51	6	-	NLGYYT	AP1T, QP4G
52	6	Ac	NLGYYT	AP1T, QP4G
53	6	Ac	NLHYYT	AP1T, QP4H
54	6	Ac	NLIYYT	AP1T, QP4I
55	6	Ac	NLKYYT	AP1T, QP4K
56	6	Ac	NLLYYT	AP1T, QP4L
57	6	Ac	NLMYYT	AP1T, QP4M
58	6	Ac	NLNYYT	AP1T, QP4N
59	6	Ac	NLPYYT	AP1T, QP4P
60	6	Ac	NLRYYT	AP1T, QP4R
61	6	-	NLSYYT	AP1T, QP4S
62	6	Ac	NLSYYT	AP1T, QP4S
63	6	Ac	NLTYYT	AP1T, QP4T
64	6	Ac	NLVYYT	AP1T, QP4V
65	6	Ac	NLWYYT	AP1T, QP4W

66	6	Ac	NLYYYT	AP1T, QP4Y
67	6	-	RLGYYA	QP4G, NP6R
68	6	Ac	RLGYYA	QP4G, NP6R
69	6	-	DLQYYA	QP6D
70	6	Ac	DLQYYA	QP6D
71	6	-	NPQYYA	LP5P
72	6	Ac	NPQYYA	LP5P
73	6	-	NFGYYA	QP4G, LP5F
74	6	-	NIGYYA	QP4G, LP5I
75	6	-	NRGYYA	QP4G, LP5R
76	6	-	NKGYYA	QP4G, LP5K
77	6	-	NDGYYA	QP4G, LP5D
78	6	Ac	RLSYYT	AP1T, QP4S, NP6R
79	6	Ac	NQSYYT	AP1T, QP4S, LP5Q
80	6	Ac	NRSYYT	AP1T, QP4S, LP5R
81	6	Ac	NKSYYT	AP1T, QP4S, LP5K
82	6	Ac	NESYYT	AP1T, QP4S, LP5E
83	6	-	NLSFYA	YP3F, QP4S
84	6	Ac	NLSFYA	YP3F, QP4S
85	6	Ac	NLSYFA	YP2F, QP4S
86	6	-	NLGYFA	YP2F, QP4G
87	6	Ac	NLGYFA	YP2F, QP4G
88	6	-	NLGFYA	YP3F, QP4G
89	6	Ac	NLGFYA	YP3F, QP4G
90	6	-	NLGHYA	YP3H, QP4G
91	6	Ac	NLGHYA	YP3H, QP4G
92	6	-	NLSHYA	YP3H, QP4S
93	6	Ac	NLSHYA	YP3H, QP4S
94	6	Ac	NLGYFT	AP1T, YP2F, QP4G
95	6	-	NLSFYT	AP1T, YP3F, QP4S
96	6	Ac	NLSFYT	AP1T, YP3F, QP4S
97	6	-	NLSYFT	AP1T, YP2F, QP4S
98	6	Ac	NLSYFT	AP1T, YP2F, QP4S
99	6	-	NLSFFT	AP1T, YP2F, YP3F, QP4S
100	6	Ac	NLSFFT	AP1T, YP2F, YP3F, QP4S
101	6	-	NLSY4T	AP1T, YP2 4, QP4S
102	6	-	NLS4YT	AP1T, YP3 4, QP4S
103	6	-	NLSY2T	AP1T, YP2 2, QP4S
104	6	-	NLSY3T	AP1T, YP2 3, QP4S
105	6	-	NLSY5T	AP1T, YP2 5, QP4S
106	6	Ac	NLSYAT	AP1T, YP2A, QP4S
107	6	Ac	NLSAYT	AP1T, YP3A, QP4S
108	6	-	NLQYHA	YP2H
109	6	-	NLAYYA	QP4A
110	6	-	NLEYYA	QP4E
111	6	-	NLLYYA	QP4L
112	6	-	NLNYYA	QP4N
113	6	-	NLPYYA	QP4P
114	6	-	NLRYYA	QP4R
115	6	-	NLTYYA	QP4T
116	6	Ac	NLSYHT	AP1T, YP2H, QP4S
117	6	Ac	NLSYWT	AP1T, YP2W, QP4S

Supplementary Table 2: Cryo-EM data collection, refinement, and validation statistics\

PDB ID	6XMJ
EMDB ID	22259
**Data collection and Processing**	
Microscope	Titan Krios
Camera	K2 Summit
Magnification	29,000
Voltage (kV)	300 keV
Total electron fluence (e^{-}/Å^2)	50
Electron flux (e^{-}/pixel/sec)	8
Defocus range (μm)	-1.5 to -3.0
Pixel size (Å)	1.03
Micrographs collected (no.)	13,392
Total extracted particles (no.)	2,650,143
Refined particles (no.)	497,630
**Reconstruction**	
Final particles (no.)	234,960
Symmetry	C1
Resolution (global, Å)	
FSC 0.5	3.1/3.0
(unmasked/masked)	3.0/2.9
FSC 0.143	2.8-3.6
(unmasked/masked)	
Resolution Range (local, Å)	
**Model composition**	
Nonhydrogen atoms	64,832
Protein residues	4,555
Ligands	0
Waters	0
**Refinement**	
MapCC (volume/masked)	0.80/0.84
Map sharpening *B* factor (Å^2)	-108
R.m.s. deviations	
Bond lengths (Å)	0.004
Bond angles (°)	0.746
**Validation**	
EMRinger score	3.97
CaBLAM outliers (%)	1.79
MolProbity score	1.13
Clashscore	1.77
Rotamer outliers (%)	0
Ramachandran plot	
Outliers (%)	0.02
Allowed (%)	3.19
Favored (%)	96.79

Supplementary Table 3. Measurements for *h*20S-PA26$^{E102A\text{-}Opt5}$ structure.

α-subunit	α1	α2	α3	α4	α5	α6	α7
Pro17 displacement (Å)	1.0	1.3	2.8	3.7	3.5	1.6	1.5
Rigid body rotation (°)	2.2	1.3	1.0	1.7	1.1	1.6	1.3
Unresolved gating residues (#)	8	7	7	2	7	3	6

C-termini (α-pocket)	α7/α1	α1/α2	α2/α3	α3/α4	α4/α5	α5/α6	α6/α7
C-tail electron density ($Å^3$)	4.7	165.8	196.8	161.8	189.0	170.2	73.9
Salt bridging distance (Å)	-	5.1	3.4	3.2	2.9	3.6	4.3
P2-P3 ϕ (°)	-	-61.5	-127.3	-121.1	-137.8	-131.8	-129.9
P2-P3 ψ (°)	-	-39.8	159.2	167.2	149.2	136.3	150.9
Tyr-Tyr π-stacking *R* (Å)	-	4.3	4.6	4.6	4.7	4.4	4.7
Tyr-Tyr π-stacking θ (°)	-	25.7	26.0	26.3	23.7	33.3	25.6

α-ring pore	α1-α4	α1-α5	α2-α5	α2-α6	α3-α6	α3-α7	α4-α7
Diameter (closed-gate) (Å) [a]	37.5	39.2	37.7	36.9	36.4	36.8	36.9
Diameter (open-gate) (Å)	41.1	41.0	40.9	41.1	41.2	41.2	41.2

R = distance between centers of aromatic rings; θ = angle of incidence formed by Tyr planes.

[a]Diameters are defined as the average distance between αPro17 Cα atoms across the α-ring denoted by the α-subunit pairs in the *h*20S (PDB ID: 4R3O [http://doi.org/10.2210/pdb4r3o/pdb]).

Supplementary Table 4. C-terminal sequences of proteasome activators from different archaeal and eukaryotic species in the UniProtKB database.

Protein	Entry	Sequence	Entry name
PAN	Q58576	LDVLYR	PAN_METJA
PAN	D4GUJ7	VSRAFA	PAN1_HALVD
PAN	Q5UT56	FTDYQY	PAN2_HALVD
PAN	Q9V2V6	DEQTEE	PSA1_HALVD
PAN	D4GYZ1	NFEGLE	PSB_HALVD
PAN	P9WQN5	NLGQYL	ARC_MYCTU
PAN	Q9HRW6	VSRTFA	PAN1_HALSA
PAN	Q9V287	HEIIYG	PAN_PYRAB
PAN	O50202	NTGQYL	ARC_RHOER
PAN	Q8TI88	ETTMFV	PAN_METAC
PAN	Q8PY58	PETMFV	PAN_METMA
PAN	Q9HNP9	YPSYIQ	PAN2_HALSA
PAN	Q9V2V5	TDEREE	PSA2_HALVD
PAN	O28303	KGVMFV	PAN_ARCFU
PAN	Q8U4H3	HEVIYG	PAN_PYRFU
PAN	O57940	HEVIYG	PAN_PYRHO
PAN	Q5JHS5	HEVMYG	PAN_THEKO
PAN	Q980M1	RREKYS	PAN_SACS2
PAN	C5A6P8	HEVMYG	PAN_THEGJ
PAN	Q975U2	RTEKYV	PAN_SULTO
PAN	Q6LWR0	LTVMYG	PAN_METMP
PAN	A9A916	LTVMYG	PAN_METM6
PAN	Q2FQ56	AGVMFA	PAN_METHJ
PAN	A6UQT3	LTTMYG	PAN_METVS
PAN	O26824	TGVMFG	PAN_METTH
PAN	A6VHR1	LTVMYG	PAN_METM7
PAN	B8GGN4	SGAMFA	PAN_METPE
PAN	C3MY47	RREKYS	PAN_SULIM
PAN	Q0W257	SGVMFA	PAN_METAR
PAN	A7I8B8	EGRMFA	PAN_METB6
PAN	Q8TX03	FKRAYH	PAN_METKA
PAN	C3N7K8	RREKYS	PAN_SULIY
PAN	Q9YAC7	TIATVI	PAN_AERPE
PAN	A4G0S4	LTVMYG	PAN_METM5
PAN	A3CV35	FGEMFA	PAN_METMJ
PAN	C3MRF1	RREKYS	PAN_SULIL
PAN	C3NFW6	RREKYS	PAN_SULIN
PAN	C4KIR6	RREKYS	PAN_SULIK
PAN	B6YXR2	HEVMYG	PAN_THEON
PAN	C3MZI6	RREKYS	PAN_SULIA
PAN	P9WQN4	NLGQYL	ARC_MYCTO
PA200	Q14997	SPCYYA	PSME4_HUMAN
PA200	Q5SSW2	SPCYYA	PSME4_MOUSE
PA200	F1MKX4	SPCYYA	PSME4_BOVIN
PA200	Q6NRP2	SPCYYA	PSME4_XENLA
PA200	F1QFR9	SPCYYA	PSM4A_DANRE
PA200	P43583	WRSYYA	BLM10_YEAST
PA200	F4JC97	SSSYFA	PSME4_ARATH
Rpt5	Q9SEI2	SLNYYA	PS6AA_ARATH
Rpt5	O76371	SLNYYA	PRS6A_CAEEL
Rpt5	Q54PN7	TLEYYA	PRS6A_DICDI
Rpt5	Q8SR13	KLLYFT	PRS6A_ENCCU
Rpt5	P17980	NLQYYA	PRS6A_HUMAN
Rpt5	O88685	NLQYYA	PRS6A_MOUSE
Rpt5	Q63569	NLQYYA	PRS6A_RAT
Rpt5	P33297	SVSFYA	PRS6A_YEAST
Rpt5	O42587	NLQYYA	PR6AA_XENLA
Rpt3	P43686	EHEFYK	PRS6B_HUMAN
Rpt3	P54775	EHEFYK	PRS6B_MOUSE

Rpt3	Q63570	EHEFYK	PRS6B_RAT
Rpt3	P33298	KFDFYK	PRS6B_YEAST
Rpt3	Q9SEI4	DFEFYK	PRS6B_ARATH
Rpt3	Q3T030	EHEFYK	PRS6B_BOVIN
Rpt3	O74894	QFAFYK	PRS6B_SCHPO
Rpt3	Q4R7L3	EHEFYK	PRS6B_MACFA
Rpt3	P46502	DFEFYK	PRS6B_CAEEL
Rpt2	P40327	LEGLYL	PRS4_YEAST
Rpt2	Q9SZD4	PEGLYM	PRS4A_ARATH
Rpt2	P62191	PEGLYL	PRS4_HUMAN
Rpt2	P62192	PEGLYL	PRS4_MOUSE
Rpt2	P48601	PEGLYL	PRS4_DROME
Rpt2	P62193	PEGLYL	PRS4_RAT
Rpt2	O16368	PEELYL	PRS4_CAEEL
Rpt2	Q90732	PEGLYL	PRS4_CHICK
Rpt2	Q8SRH0	SAGLYS	PRS4_ENCCU

Supplementary Table 5. List of primer sequences used to generate plasmids.

Primer name	Gene and description of cloning	Primer sequence (5' to 3')
KO1	PA26 Deactivation (E102A) forward	CATAAGGAAGCAGATAACCTTGGTGTG
KO2	PA26 Deactivation (E102A) reverse	CTCGGGGATGCGAATTGC
KO3	LIC PA26 forward	TACTTCCAATCCAATGCAATGCCACCGAAACGCGCCGC
KO4	LIC PA26 m1 (NLQYYA) reverse	TTATCCACTTCCAATGTTACGCATAGTATTGCAAATTTCCCGTACGAGGCTGAATGA
KO5	LIC PA26 m2 (NLSYYT) reverse	TTATCCACTTCCAATGTTATGTGTAATACGACAGGTTTCCCGTACGAGGCTGAATGA
KO6	LIC PA26 m3 (NLSYFT) reverse	TTATCCACTTCCAATGTTAGGTAAAGTACGATAAATTTCCCGTACGAGGCTGAATGA
KO7	PA26 m2 to m4 (NLSFYT) forward	AACCTGTCGTTTTACACATAACATTGG
KO8	PA26 m3 to m5 (NLSFFT) forward	AATTTATCGTTCTTTACCTAACATTGGAAGTG
KO9	PA26 m2 to m6 (NLSYAT) forward	AACCTGTCGTATGCCACATAACATTGG
KO10	PA26 m2 & m3 reverse	TCCCGTACGAGGCTGAAT
KO11	PA26 m2 to m7 (NLSAYT) forward	GGAAACCTGTCGGCTTACACATAACATTGG
KO12	PA26 m2 to m7 reverse	CGTACGAGGCTGAATGAGC
KO13	LIC MBP forward	TACTTCCAATCCAATGCAGGAGATATACCATGGG
KO14	LIC MBP-2 (NLSYYT) reverse	TTATCCACTTCCAATGTTAAGTATAGTAGGAAAGATTGGATTGGAAGTACAGG
KO15	LIC MBP-3 (NLSYFT) reverse	TTATCCACTTCCAATGTTACGTGAAATAGGACAAGTTGGATTGGAAGTACAGG
KO16	LIC MBP-4 (NLSFYT) reverse	TTATCCACTTCCAATGTTAGGTATAAAAGGATAAATTGGATTGGAAGTACAGG
KO17	LIC MBP-5 (NLSFFT) reverse	TTATCCACTTCCAATGTTATGTAAAAAACGATAAGTTGGATTGGAAGTACAGG
KO18	MBP-3 to MBP-6 (NLSYAT) forward	TCTTTCTACGCTACTTAACATTGGAAGTG
KO19	MBP-3 to MBP-6 (NLSYAT) reverse	TTGGATTGGAAGTACAGG
KO20	MBP-3 to MBP-7 (NLSAYT) forward	CAATCTTTCCGCCTATACTTAACATTGGAAG
KO21	MBP-3 to MBP-7 (NLSAYT) reverse	GATTGGAAGTACAGGTTTTC
