# Supplementary material for: The YΦ motif defines the structure-activity relationships of human 20S proteasome activators
Source: Nat Commun. 2022 Mar 9;13:1226. doi: 10.1038/s41467-022-28864-x (PMC8907193; doi:10.1038/s41467-022-28864-x)
Supplement: Supplementary file 2 — Reporting Summary [file 41467_2022_28864_MOESM2_ESM.pdf]

nature research

Corresponding author(s): Gestwicki, Jason E

Last updated by author(s): May 25 2021

# Reporting Summary

Nature Research wishes to improve the reproducibility of the work that we publish. This form provides structure for consistency and transparency in reporting. For further information on Nature Research policies, see our Editorial Policies and the Editorial Policy Checklist.

## Statistics

For all statistical analyses, confirm that the following items are present in the figure legend, table legend, main text, or Methods section.

| n/a | Confirmed | |
|---|---|---|
| ☐ | ☒ | The exact sample size (*n*) for each experimental group/condition, given as a discrete number and unit of measurement |
| ☐ | ☒ | A statement on whether measurements were taken from distinct samples or whether the same sample was measured repeatedly |
| ☐ | ☒ | The statistical test(s) used AND whether they are one- or two-sided<br>*Only common tests should be described solely by name; describe more complex techniques in the Methods section.* |
| ☐ | ☒ | A description of all covariates tested |
| ☐ | ☒ | A description of any assumptions or corrections, such as tests of normality and adjustment for multiple comparisons |
| ☐ | ☒ | A full description of the statistical parameters including central tendency (e.g. means) or other basic estimates (e.g. regression coefficient) AND variation (e.g. standard deviation) or associated estimates of uncertainty (e.g. confidence intervals) |
| ☐ | ☒ | For null hypothesis testing, the test statistic (e.g. *F*, *t*, *r*) with confidence intervals, effect sizes, degrees of freedom and *P* value noted<br>*Give P values as exact values whenever suitable.* |
| ☒ | ☐ | For Bayesian analysis, information on the choice of priors and Markov chain Monte Carlo settings |
| ☒ | ☐ | For hierarchical and complex designs, identification of the appropriate level for tests and full reporting of outcomes |
| ☒ | ☐ | Estimates of effect sizes (e.g. Cohen's *d*, Pearson's *r*), indicating how they were calculated |

*Our web collection on statistics for biologists contains articles on many of the points above.*

## Software and code

Policy information about availability of computer code

| | |
|---|---|
| Data collection | Activation information was collected using Molecular Devices SoftMax Pro 6.5.1 and raw data exported as txt files. Binding information was collected using Octet RED384 and raw and analyzed data exported as csv files. Cryo-EM data was collected using Leginon 3.3 |
| Data analysis | Data was analyzed in Microsoft Excel 16.16.23, GraphPad PRISM 8.1.2, and Octet Data Analysis 7.1, using standard algorithms. Cryo-EM image processing was done with Appion 3.3, MotionCor2, CTFFind4, Relion 3.0, CryoSPARC, Bsoft 1.9.0, gCTF, Phenix 1.10.1, Coot 0.8.9.1, EMAN 1.9, ChimeraX 1.14 and PyMOL 2.3.2. |

For manuscripts utilizing custom algorithms or software that are central to the research but not yet described in published literature, software must be made available to editors and reviewers. We strongly encourage code deposition in a community repository (e.g. GitHub). See the Nature Research guidelines for submitting code & software for further information.

## Data

Policy information about availability of data

All manuscripts must include a data availability statement. This statement should provide the following information, where applicable:
- Accession codes, unique identifiers, or web links for publicly available datasets
- A list of figures that have associated raw data
- A description of any restrictions on data availability

The atomic coordinates for the PA26(E102A)-Opt5-bound human 20S (single-capped) structure has been deposited in the Protein Data Bank (PDB) under accession code 6XMJ. The corresponding EM density maps have been deposited to the Electron Microscopy Data Bank under accessions EMD-22259. All data used in the figures is available on request.

# Field-specific reporting

Please select the one below that is the best fit for your research. If you are not sure, read the appropriate sections before making your selection.

☒ Life sciences ☐ Behavioural & social sciences ☐ Ecological, evolutionary & environmental sciences

For a reference copy of the document with all sections, see nature.com/documents/nr-reporting-summary-flat.pdf

# Life sciences study design

All studies must disclose on these points even when the disclosure is negative.

| | |
|---|---|
| Sample size | Sample sizes were selected to be inclusive of typical variance in biochemical measurements (enzyme turnover and protein-protein binding), including multiple, independent tests (biological replication) and multiple tests on the same day (technical replicates). The N values, with a breakdown of the biological/technical replication are included in each figure legend. |
| Data exclusions | No data was excluded from the analyses. All replicates were used. |
| Replication | Each experiment was repeated through independent biological replications, as noted in the figure legends. |
| Randomization | Randomization was not used in these experiments, due to the availability of experimental controls, such as solvent only treatments and systematic structural variations of each peptide in a series. |
| Blinding | Blinding was not relevant to this study, due to the availability of experimental controls. |

# Reporting for specific materials, systems and methods

We require information from authors about some types of materials, experimental systems and methods used in many studies. Here, indicate whether each material, system or method listed is relevant to your study. If you are not sure if a list item applies to your research, read the appropriate section before selecting a response.

## Materials & experimental systems

| n/a | Involved in the study | |
|---|---|---|
| ☒ | ☐ | Antibodies |
| ☒ | ☐ | Eukaryotic cell lines |
| ☒ | ☐ | Palaeontology and archaeology |
| ☒ | ☐ | Animals and other organisms |
| ☒ | ☐ | Human research participants |
| ☒ | ☐ | Clinical data |
| ☒ | ☐ | Dual use research of concern |

## Methods

| n/a | Involved in the study | |
|---|---|---|
| ☒ | ☐ | ChIP-seq |
| ☒ | ☐ | Flow cytometry |
| ☒ | ☐ | MRI-based neuroimaging |
